# A Case Report of Dental Management of Patient With Arnold Chiari I Malformation

**DOI:** 10.1155/crid/2868368

**Published:** 2025-04-29

**Authors:** Amani M. Harrandah

**Affiliations:** Department of Basic and Clinical Oral Sciences, Umm Al-Qura University College of Dental Medicine, Makkah, Saudi Arabia

## Abstract

Arnold Chiari malformation (CM) is a neurological disorder marked by the downward displacement of the cerebellar tonsils through the foramen magnum into the spinal canal, impacting the brain's posterior fossa, often associated with myelomeningocele, a form of spina bifida where the spinal cord protrudes through a spinal defect. Diagnosis is commonly made using MRI, with surgical intervention sometimes needed to relieve pressure on the brain and spinal cord. There are six types of CMs; Type I (CM1) is the most prevalent and is often identified in adulthood. CM1 involves herniation of the cerebellar tonsils through the foramen magnum, potentially causing symptoms such as headaches exacerbated by coughing, neck pain, dizziness, and sensory disturbances. Severe cases may include dysphagia, motor control issues, and sleep apnea. In dental treatment, CM patients should avoid prolonged neck hyperextension, which can increase discomfort, and metal appliances may need removal before MRI imaging. This case report documents the first case of CM1 at Umm Al-Qura University Dental Teaching Hospital and describes a tailored dental treatment plan designed to address the patient's specific needs.

## 1. Introduction

Arnold Chiari syndrome, or Arnold Chiari malformation (CM), is indeed a notable neurological condition. It refers to a group of congenital malformations primarily affecting the brain's posterior fossa, which includes the hindbrain (the cerebellum and brainstem). CMs involve the downward displacement of the cerebellar tonsils through the foramen magnum—the opening at the base of the skull—into the spinal canal. CM is often associated with myelomeningocele, a severe form of spina bifida, in which the spinal cord and its coverings protrude through a defect in the spine [1].

CMs were first described by Hans Chiari in 1890, and the term “Arnold Chiari” is sometimes used in reference to Julius Arnold, who contributed to the description of the malformation's association with spinal cord defects like myelomeningocele. The condition is typically diagnosed using MRI, and treatment can involve surgery to relieve pressure on the brain and spinal cord, especially in more severe cases [2].

There are six different types of CMs, with Type I (CM1) being the most common and often diagnosed later in life, while Types II, III, and IV are more severe and usually diagnosed at birth or early infancy [3]. CM1 involves the downward displacement of the cerebellar tonsils, along with slight elongation of the fourth ventricle and the medulla oblongata. Chiari Type II, on the other hand, is characterized by the descent of the cerebellar vermis, accompanied by the downward displacement of the fourth ventricle and CM1 lower brainstem [4, 5].

CM1, by far the most common type, is a condition where the cerebellar tonsils herniate through the foramen magnum, which can lead to a variety of symptoms due to the disruption of cerebrospinal fluid (CSF) flow and pressure on surrounding neural structures. The most common symptoms include headaches, typically worsened by coughing, sneezing, or straining. Other common manifestations include neck pain, dizziness, unsteady gait, and coordination issues, as well as sensory disturbances such as numbness and tingling in the extremities. Some patients may also experience more severe neurological symptoms such as difficulty swallowing (dysphagia), impaired fine motor control, and in some cases breathing difficulties such as sleep apnea. Furthermore, conditions like syringomyelia, where a fluid-filled cyst forms within the spinal cord, often accompany CM1, further complicating the clinical picture. While many individuals with CM1 are asymptomatic, those with progressive symptoms may require surgical intervention to relieve pressure and restore CSF flow [6–8].

Patients with CM1 may present with symptoms that mimic TMJ disorders, such as facial pain and masticatory muscle discomfort. This overlap can complicate accurate diagnosis and delay appropriate management. The facial pain observed in ACM I is often attributed to increased intracranial pressure affecting the trigeminal nerve tract, leading to sensations that resemble TMJ dysfunction [9]. Furthermore, trigeminal neuralgia, characterized by sudden, severe facial pain, has also been linked to CM1. The exact mechanism remains unclear, but proposed explanations include vascular compression at the nerve root entry zone and direct brainstem compression due to the malformation [9, 10].

During dental treatment, patients with CM should avoid extended periods of neck hyperextension while seated in the dentist's chair, as this can worsen their symptoms. Additionally, fixed metal appliances like braces may need to be postponed or removed if an MRI scan is required.

The purpose of this study was to report the first case of CM1 visiting Umm Al-Qura University dental teaching hospital and discuss dental treatment plan for this patient.

## 2. Case Presentation

**Figure 1:**
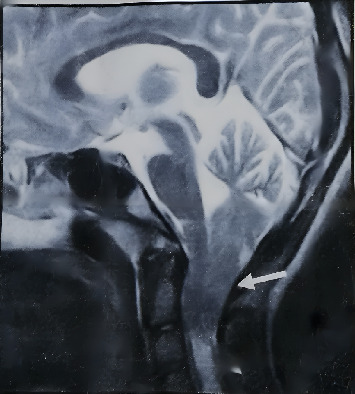
MRI of the brain. MRI of the brain showing downward displacement of the cerebellar tonsils below the foramen magnum, resulting in narrowing and compression of the craniocervical junction and upper spinal cord.

A 20-year-old female presented to the Umm Al-Qura University Dental Teaching Hospital in Makkah, Saudi Arabia, seeking comprehensive dental treatment and restorative care. During the medical history assessment, the patient was identified as having CM1, characterized by the downward displacement of the cerebellar tonsils below the foramen magnum, resulting in narrowing and compression of the craniocervical junction and upper spinal cord. This condition was associated with syrinx formation in the upper cord, as evidenced by a central CSF intramedullary lesion (Figure 1). The patient had undergone posterior fossa decompression surgery to alleviate pressure on the medulla oblongata as part of her medical management.

Additionally, the patient reported symptoms of lower limb weakness, visual disturbances, and difficulty swallowing. The patient has no reported history of orofacial pain, temporomandibular joint dysfunction, or trigeminal neuralgia. A comprehensive treatment plan was developed, and informed consent was obtained for each step of the proposed dental procedures, including consent for the publication of clinical photographs documenting the treatment process. Ethical approval for the case report was granted by the Institutional Review Board (IRB) of Umm Al-Qura University.

### 2.1. Treatment Plan

#### 2.1.1. Phase 1: Initial Assessment and Pain Management

**Figure 2:**
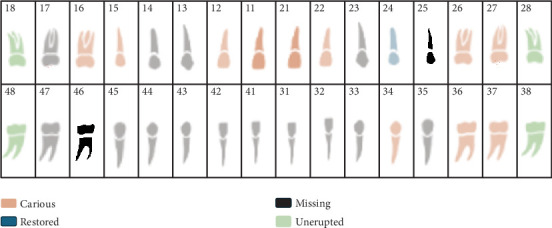
Dental chart. Detailed dental chart showing missing teeth (46, 25), carious teeth (16, 15, 12, 11, 21, 22, 26, 27, 34, 36, 37), unerupted teeth (18, 28, 38, 48), and restored tooth (24).

**Figure 3:**
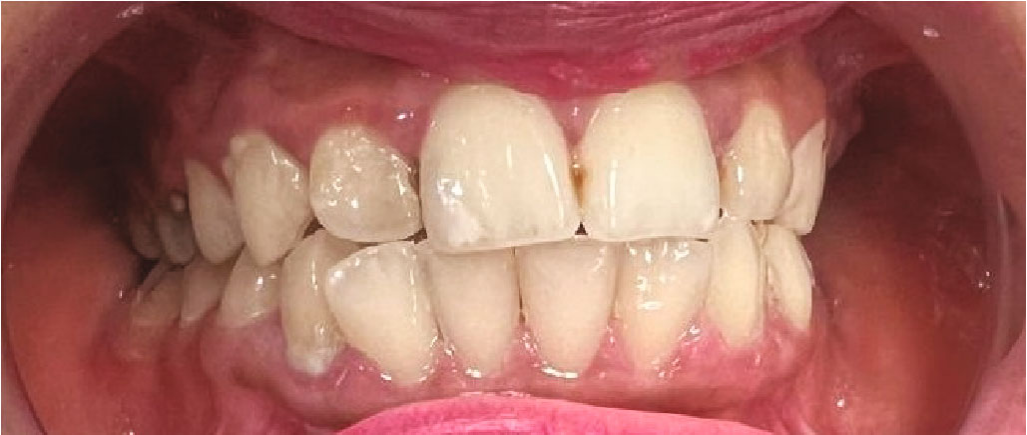
Anterior view of the teeth. Anterior image of the patient' anterior teeth.

During the oral examination, the patient presented with multiple carious teeth, residual roots, and missing teeth. Detailed dental chart is presented in Figure 2. The soft tissues appeared within normal limits, with a moderate accumulation of supra- and subgingival plaque and calculus. Additionally, the patient exhibited limited mouth opening, which may be associated with her underlying medical condition. Due to this limitation, comprehensive full-mouth intraoral images were not feasible; instead, only an anterior view of the teeth was captured (Figure 3).

**Figure 4:**
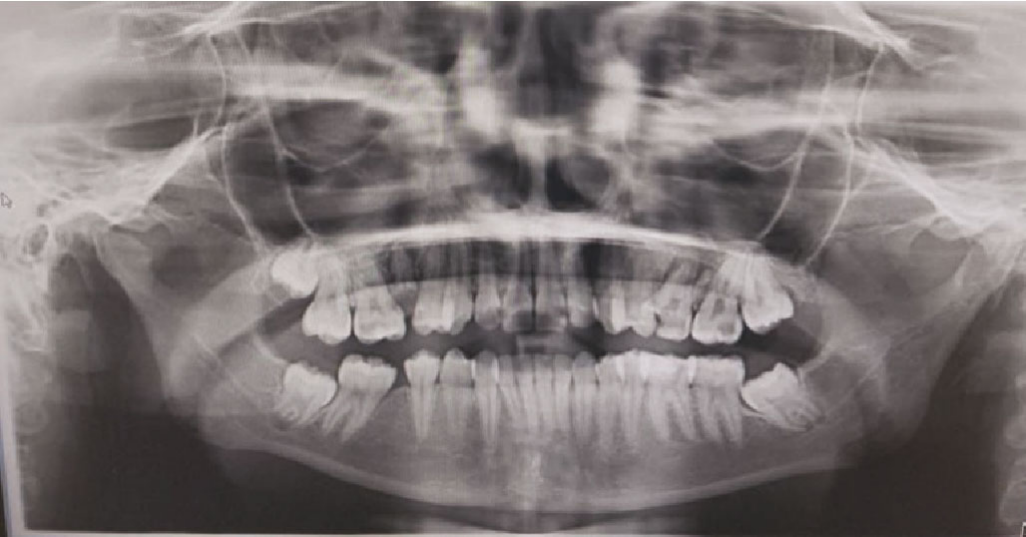
Radiographic assessment of the teeth. Panoramic x-ray of the teeth showing significant bone loss, residual roots, and missing and carious teeth.

Radiographic evaluation (Figure 4) revealed significant bone loss, residual roots, and missing teeth. Given the patient's specific needs, several modifications were implemented to ensure her comfort during the assessment. The patient expressed the need for frequent breaks during treatment and reported intolerance to reclined dental chair positions. To address this, a semisupine or upright chair position was utilized during examinations to reduce neck strain and enhance patient comfort. Additional breaks were provided throughout the procedures, and alternative imaging techniques were employed during radiographic assessment to minimize discomfort.

A comprehensive treatment plan was developed based on the patient's clinical findings, medical condition, and radiographic evaluation. Adjustments were made to accommodate the patient's specific requirements, ensuring both her comfort and the efficacy of the planned dental procedures (Table 1).

#### 2.1.2. Phase 2: Disease Control

All carious lesions on teeth (16, 15, 12, 11, 21, 22, 26, 27, 34, 36, 37) were removed, and temporary restorations were placed. Due to neurological involvement, patients with CM1 may have heightened sensitivity to pain, and dental procedures may exacerbate headache symptoms; thus, during this phase, shorter appointments were considered to reduce the risk of triggering pain or discomfort. Also, local anesthesia and pain killers were considered during the treatment. CM1 patients may have difficulty swallowing or impaired airway reflexes, increasing their risk of aspiration; therefore, high-power surgical suction was used to reduce the risk of aspiration (Table 1).

#### 2.1.3. Phase 3: Extraction of Nonrestorable Teeth and Root Debridement

Tooth Numbers 15 (remining root) and 26 (nonrestorable tooth) were extracted. Shorter appointments were considered during this phase where each tooth was extracted in a different appointment. Also, using appropriate local anesthesia and pain killers was considered to reduce pain during and after the extraction. Furthermore, during extraction, the dental chair was in an upright position to reduce pressure on the neck.

#### 2.1.4. Phase 4: Restoration of Restorable Teeth

Composite restoration were placed on Tooth Numbers 11, 12, 21, 24, 27, 34, 37, and 46. Crown preparation was done for Teeth 16, 17, and 36. Due to multiple restorations and crown and longer appointment might not be very comfortable for the patient, each tooth was done in a separate appointment.

#### 2.1.5. Phase 5: Replacement of Missing Teeth

Since the space is lost, replacing missing teeth needs extensive surgical and orthodontic treatment. Due to the patient condition where long appointments are not well tolerated, implants were not considered for this patient.

#### 2.1.6. Phase 6: Maintenance and Follow-Up

Finally, in the last phase, follow-up appointments every 6 months were scheduled, and oral hygiene practices were reinforced.

Detailed modification of the treatment plan in each phase is described in Table 1.

In conclusion, patients with CM1 may experience headaches, neck pain, and muscle weakness, which can make dental chair positioning challenging. Thus, modifications in the treatment should be considered according to the patient's need. These modifications include using a semisupine or upright position to reduce pressure on the neck and improve comfort and ensuring that the head is well supported and avoid hyperextension of the neck to minimize discomfort or aggravation of symptoms. Furthermore, due to neurological involvement, patients with CM1 may have heightened sensitivity to pain, and since dental procedures may exacerbate headache symptoms, shorter appointments were considered to reduce the risk of triggering pain or discomfort while using local anesthesia effectively and monitor the patient's response. Moreover, any procedures that require the patient to hold their mouth open for extended periods were avoided as this can strain the neck and cervical spine.

In addition to modifications in the treatment plan to avoid any pain or discomfort, since patients with CM1 experience anxiety about exacerbating their symptoms or being in uncomfortable positions, reassurance was provided to the patient, with frequent breaks, and open communications to reduce anxiety.

## 3. Discussion

CM1 is a unique clinical condition requiring specific modifications in dental care due to the neurological symptoms associated with the malformation, such as headaches, neck pain, and muscle weakness. These symptoms are known to intensify with neck hyperextension and prolonged periods of immobility, both common in dental settings [1, 6]. For this patient, modifications were made to accommodate her comfort, such as using a semisupine or upright position and providing head support, which aligns with best practices in CM1 management.

The use of shorter dental appointments also addresses the increased pain sensitivity seen in CM1 patients [8]. By minimizing the appointment length, potential exacerbations of headache symptoms were reduced, improving the patient's overall experience. Additionally, utilizing local anesthesia helped manage pain effectively without causing excessive sedation, a crucial consideration given that some CM1 patients may exhibit heightened sensitivity to pain due to neurological involvement.

The need to avoid extended periods with the mouth open was another essential modification to prevent strain on the cervical spine. This approach aligns with reports emphasizing the vulnerability of CM1 patients to neck discomfort due to cervical stress [2, 3]. The emphasis on shorter procedures and ergonomic positioning underscores the necessity of customized care strategies in this population.

This case highlights the importance of an interdisciplinary approach, where dental and medical teams collaborate to optimize care for patients with neurological conditions like CM1. As emphasized in current literature, integrating modifications that consider the neurological state of the patient can significantly impact the success of the treatment and patient satisfaction [5, 7]. This approach allows for both effective dental management and improved quality of life for individuals with CM1, supporting the notion that dental care can be both safe and comfortable for these patients.

## 4. Conclusion

In conclusion, patients with CM1 need a tailored, empathetic approach to dental care, with special attention to neck support, pain management, and minimizing strain on the cervical spine. Coordination with the patient's medical team can ensure safe and effective dental care, helping to manage both their oral health and neurological well-being.

## Figures and Tables

**Table 1:** A comprehensive treatment plan, incorporating modifications to address the patient's unique needs and specific requirements.

**Phase**	**Recommended treatment plan**	**Modification according to CM1 patient**
Phase 1: Initial assessment and pain management	Taking medical and dental history Full-mouth examination with visual and radiographic assessment Address any acute pain or infection first	Shorter appointments with faster thorough examination to reduce the risk of triggering pain or discomfort Use a semisupine or upright position during examination to reduce pressure on the neck and improve comfort Multiple breaks during examination Careful positioning and using alternative imaging technique during radiographic imaging to avoid discomfort

Phase 2: Disease control Oral hygiene instructions	Educate the patient on proper oral hygiene practices and dietary modifications Caries removal for all carious teeth (16, 15, 12, 11, 21, 22, 26, 27, 34, 36, 37) and temporary restorations	Due to neurological involvement, patients with CM1 may have heightened sensitivity to pain, and dental procedures may exacerbate headache symptoms; thus, shorter appointments were considered to reduce the risk of triggering pain or discomfort Using local anesthesia and pain killers considered as well as monitor the patient's response High-power surgical suction was used to reduce the risk of aspiration

Phase 3: Extraction of nonrestorable teeth and root debridement	Extraction of 15 (remining root) and 26 (nonrestorable tooth)	Shorter two separate appointments were booked to avoid triggering pain and discomfort Appropriate local anesthesia and pain killers considered to reduce pain during and after the extraction

Phase 4: Restoration of restorable teeth	Restorations for all carious teeth Crown preparation for teeth that have lost substantial structure due to caries	Shorter appointment was booked to avoid triggering pain and discomfort

Phase 5: Replacement of missing teeth	Replacement of the missing teeth would require extensive surgical and orthodontic interventions due to the loss of space. However, considering the patient's condition and inability to tolerate prolonged appointments, implant placement was deemed unsuitable for this case	NA

Phase 6: Maintenance and follow-up	Schedule follow-up appointments every 3–6 months and reinforce oral hygiene practices	NA

## Data Availability

Data sharing is not applicable to this article as no new data were created or analyzed in this study.

## References

[1] Pearce J. M. (2000). Arnold Chiari, or “Cruveilhier Cleland Chiari” Malformation. *Journal of Neurology, Neurosurgery, and Psychiatry*.

[2] Soleau S., Tubbs R. S., Oakes W. J. (2008). Chiari Malformations. *Principles and Practice of Pediatric Neurosurgery*.

[3] Vannemreddy P., Nourbakhsh A., Willis B., Guthikonda B. (2010). Congenital Chiari Malformations. *Neurology India*.

[4] Caviness V. S. (1976). The Chiari Malformations of the Posterior Fossa and Their Relation to Hydrocephalus. *Developmental Medicine and Child Neurology*.

[5] Piper R. J., Pike M., Harrington R., Magdum S. A. (2019). Chiari Malformations: Principles of Diagnosis and Management. *BMJ*.

[6] Fric R., Eide P. K. (2020). Chiari Type 1–A Malformation or a Syndrome? A Critical Review. *Acta Neurochirurgica*.

[7] Zisakis A., Sun R., Pepper J., Tsermoulas G., Rocco C. (2023). Chiari Malformation Type 1 in Adults. *Advances and Technical Standards in Neurosurgery*.

[8] Valentini L. G., Galbiati T. F., Saletti V., Visocchi M. (2023). Evaluation of Adult and Pediatric Chiari Type 1 Malformation Patients: Do Consensus Documents Fit Everyday Practice?. *The Funnel: From the Skull Base to the Sacrum: New Trends, Technologies and Strategies*.

[9] de Arruda J. A., Figueiredo E., Monteiro J. L., Barbosa L. M., Rodrigues C., Vasconcelos B. (2018). Orofacial Clinical Features in Arnold Chiari Type I Malformation: A Case Series. *Journal of Clinical and Experimental Dentistry*.

[10] Dipalma G. (2022). Oro-Facial Pain in Chiari Type 1 Malformation. *Journal of Biological Regulators & Homeostatic Agents*.

